# Dermatomyositis in a COVID-19 positive patient

**DOI:** 10.1016/j.jdcr.2021.04.036

**Published:** 2021-05-26

**Authors:** Bao Vincent K. Ho, Edward W. Seger, Kaitlyn Kollmann, Anand Rajpara

**Affiliations:** a Division of Dermatology, University of Kansas Medical Center, Kansas City, Kansas; b School of Medicine, University of Kansas Medical Center, Kansas City, Kansas

**Keywords:** COVID-19, dermatology, dermatomyositis, myositis, neuromuscular

## Introduction

Cutaneous manifestations of COVID-19 are often atypical and nonspecific, which raises the question whether a link exists between skin manifestations and the virus. The most common and widely reported finding during or following COVID-19 is the development of chilblain-like lesions; however, this remains a contentious topic.^1^ A number of neuromuscular manifestations of COVID-19 have also been reported such as myalgia, critical-illness myopathy and neuropathy.^2^ The development of autoimmune sequelae during or following COVID-19 is sparsely reported, with only sporadic cases of autoimmune myositis, Kawasaki disease in children, and rheumatic disease.^3^^,^^4^ The Center for Disease Control has newly recognized multi-system inflammatory syndrome in adults as an emerging association with COVID-19, for which dermatologic findings were the presenting symptoms in recently reported cases.^5^

The association between COVID-19 and the development of multi-system autoimmune disorders remains unclear; however, it has been suggested that viral infections may serve as a trigger. Given the expanding list of long-term sequelae, which may develop following COVID-19, it is important to report unique presentations in order to aid clinicians in prompt diagnosis. We present a case of a patient who developed dermatomyositis following COVID-19.

## Case report

A 58-year-old previously healthy Hispanic man was diagnosed by polymerase chain reaction with symptomatic SARS-CoV-2 infection (including fever, diaphoresis, and chills), which did not require hospitalization, and which spontaneously improved with symptomatic treatment over several days. Following this initial improvement, he began to develop fatigue, myalgias, and generalized muscle weakness, which was most severe in the proximal parts of his lower extremities. These symptoms gradually worsened over a period of 1 month and were accompanied by an unintentional 30-pound weight loss and progressive shortness of breath, which prompted his presentation at our emergency department. He denied trauma or use of new medications or substances preceding or during these symptoms.

**Fig 1:**
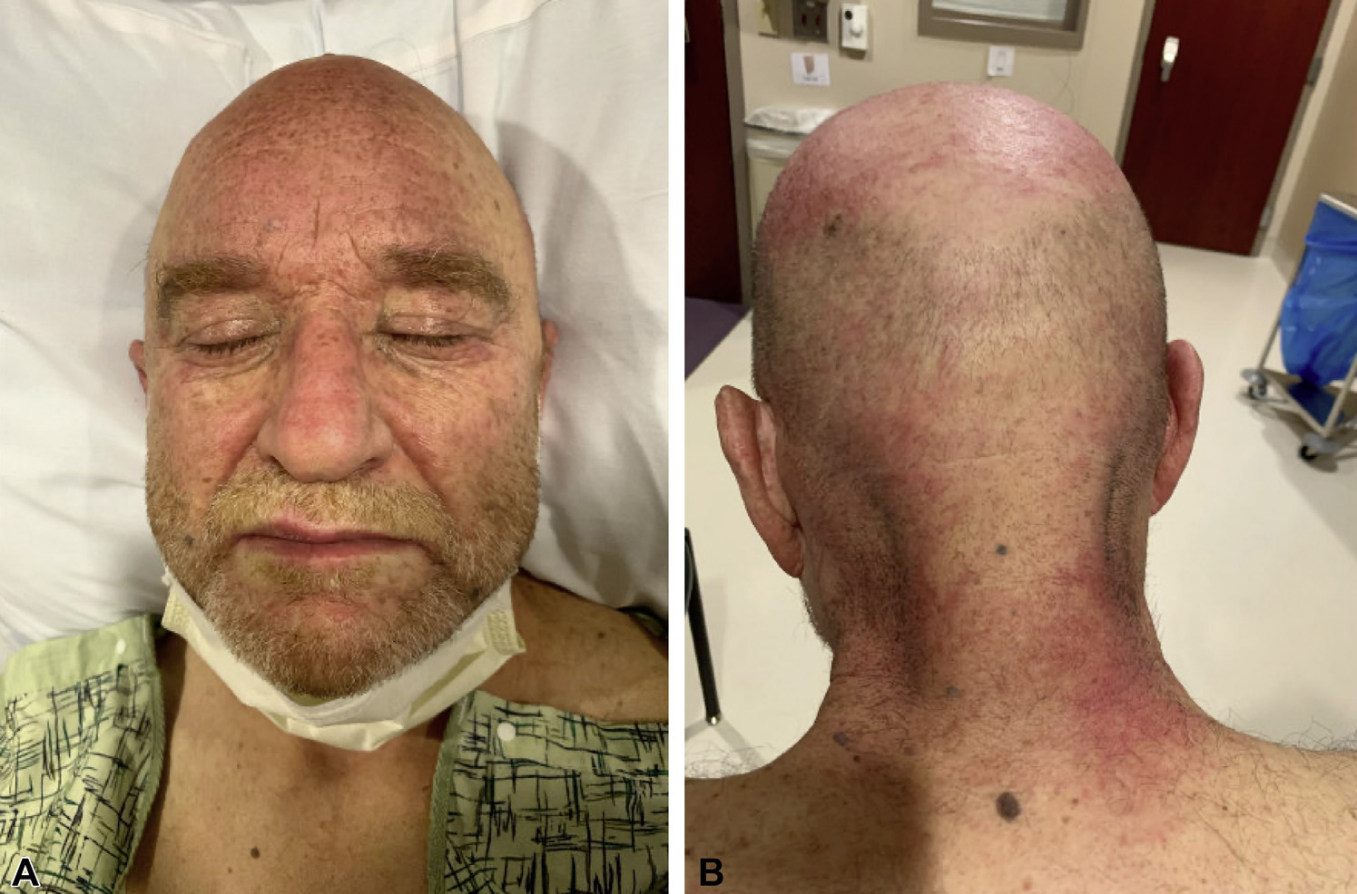
Erythematous papules coalescing into plaques on the (**A**) forehead, eyelids, cheeks, nose, and **(B)** posterior part of the neck.

**Fig 2:**
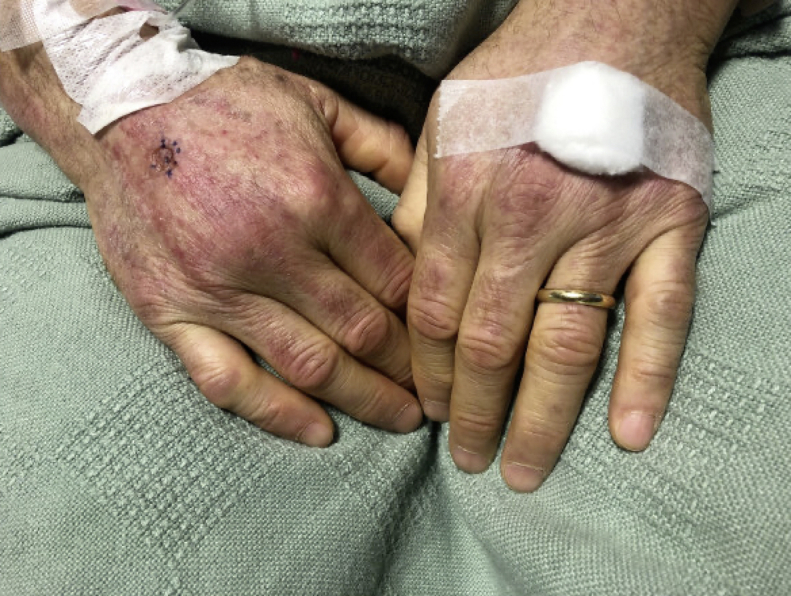
Violaceous patches over the dorsal aspects of the hands, where a shave biopsy was obtained.

Dermatology was consulted for the simultaneous development of an asymptomatic erythematous eruption beginning on his scalp, then spreading to the face and posterior part of the neck, which began during the time of his COVID-19 diagnosis 1 month previously. On presentation, erythematous papules coalescing into plaques were noted on the scalp, neck, forehead, eyelids, cheeks, nose (Fig 1, *A*), and posterior part of the neck (Fig 1, *B*), as well as violaceous patches over the dorsal aspects of the hands (Fig 2). Musculoskeletal examination was significant for mild upper extremity flexor weakness, and the patient was unable to raise his hands above his head. Normal strength and mobility were noted on the lower extremities, and patient denied paresthesia. Otolaryngology was consulted for worsening odynophagia; however, no anatomic abnormalities were visualized by fiberoptic scope.

A computed tomography scan of the chest obtained to exclude pulmonary embolism showed patchy ground-glass opacities, compatible with COVID-19 along with a pulmonary embolus in a subsegmental left lower lobe. The laboratory results revealed mildly elevated aspartate aminotransferase (313 U/L) and alanine aminotransferase (117 U/L), but otherwise revealed an unremarkable complete blood count and general biochemistry panel. Serum levels of creatine kinase (9684 U/L), aldolase (52.6 U/L), and D-dimer (1446 ng/mL) were markedly elevated. The results of an extensive autoimmune and infectious disease workup, including antinuclear antibody (titer <1:80) and anti-SARS-CoV-2 antibody tests, were unrevealing. The myositis panel (MyoMarker Panel 3; Mayo Clinic Laboratories) including anti-Jo-1, anti-U1-RNP, and anti-Mi-2 antibodies was also unremarkable.

**Fig 3:**
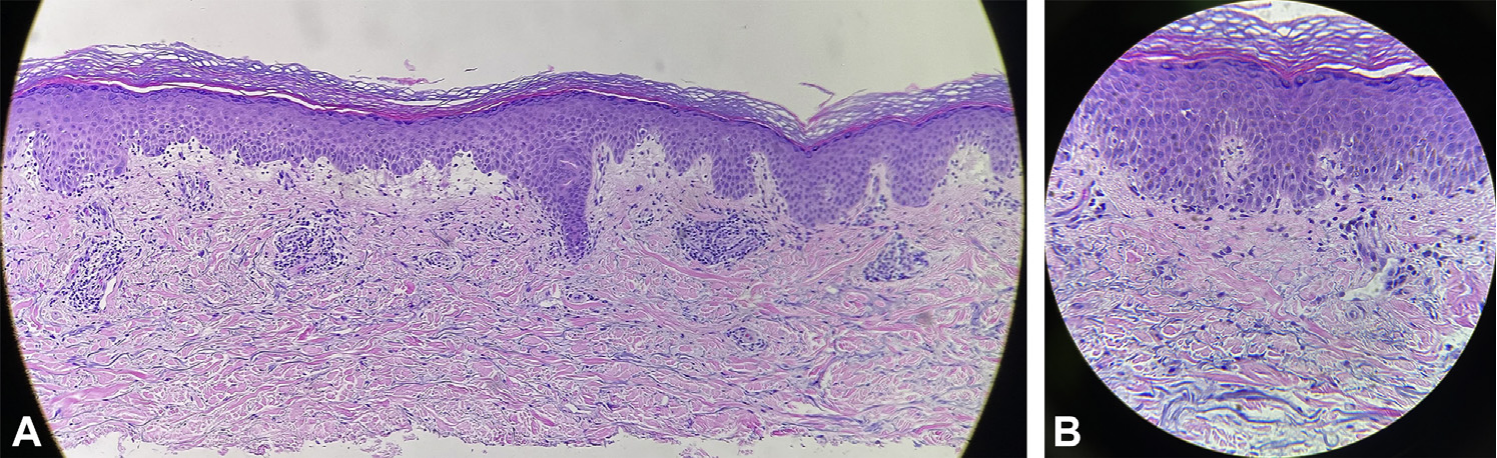
Shave biopsy obtained from the dorsal aspect of the patient's hand demonstrating vacuolar interface changes with subepidermal edema and perivascular mixed cell infiltrate, findings compatible with connective tissue diseases, such as dermatomyositis (Hematoxylin-eosin stain; original magnifications: **A**, ×10; **B** ×40.

A shave biopsy of the dorsal aspect of his right hand showed vacuolar interface changes with subepidermal edema and perivascular mixed cell infiltrate (Fig 3). Electromyography showed evidence of myopathy with mild irritative features. A biopsy of his right biceps muscle showed mild perifascicular atrophy, but no evidence of necrosis, regeneration, or inflammation. These clinical and pathologic findings were consistent with dermatomyositis.

The patient was treated with intravenous methylprednisolone 500 mg twice daily for 5 days, which was transitioned to oral prednisone 60 mg for 1 month and 40 mg daily for 1 month thereafter, along with methotrexate 10 mg weekly. Cutaneous manifestations were treated with topical triamcinolone ointment to affected areas on the body and hydrocortisone ointment for the face. Laboratory studies carried out during inpatient stay showed improvement in liver enzyme levels, CK, and aldolase, with subsequent levels normalized at 1-month outpatient follow-up. The patient experienced improved muscle strength and was ultimately discharged 3 days after beginning oral steroids. Following discharge, a computed tomography scan of the abdomen and pelvis was obtained for internal malignancy screening, which were negative. Additional studies to rule out malignancy were deferred for outpatient rheumatology follow-up.

## Discussion

Dermatomyositis has rarely been associated with infections, with few reported cases after Epstein-Barr virus infection.^6^ Similar to many other autoimmune diseases, environmental factors or drugs appear to trigger the onset of disease in some cases. This is the first reported case of new-onset dermatomyositis after COVID-19 infection in the United States.

Patients presenting with potentially COVID-19-associated cutaneous manifestations may demonstrate a spectrum of illness severity. Studies suggest that skin findings reported in SARS-CoV-2-infected patients are nonspecific and cannot be considered as diagnostic tools for establishing a diagnosis of COVID-19.^7^ However, dermatologists confronted with skin lesions in patients with suspected or confirmed SARS-CoV-2 infection should be aware of cutaneous manifestations that can be indicative of secondary systemic involvement.

It is also clinically important to carefully distinguish between interstitial lung disease attributed to connective tissue disease and COVID-19, as certain myositis-specific autoantibodies associated with dermatomyositis have been associated with significant morbidity from rapid lung damage progression.^8^ Therefore, repeated testing for SARS-CoV-2 is necessary, and serologic markers and identification of characteristic rashes of dermatomyositis may help provide diagnostic clues for prompt identification and treatment.

While several cutaneous findings are widely reported in association with COVID-19, the disproportionate media and academic attention that these manifestations receive may overlook the wide variety of dermatologic findings that may present. The pathogenesis of COVID-19-related dermatomyositis has not been well studied. Recent identification of 3 immunogenic epitopes found in SARS-CoV-2-positive patients with autoimmune dermatomyositis could suggest an overlapping mechanism for immune-pathogenesis.^9^ This current report highlights that dermatologists should be wary of viral triggers, including SARS-CoV-2, as to the cause of dermatomyositis, which at this time remains largely unknown.

## Conflicts of interest

None disclosed.
